# Application of respiratory motion management technology for patients with lung cancer treated with stereotactic body radiotherapy (Review)

**DOI:** 10.3892/ol.2025.15161

**Published:** 2025-06-27

**Authors:** Kainan Shao, Weijun Chen, Yaping Xu, Shuangyan Yang

**Affiliations:** 1 Cancer Center, Department of Radiation Oncology, Zhejiang Provincial People's Hospital (Affiliated People's Hospital), Hangzhou Medical College, Hangzhou, Zhejiang 310014, P.R. China; 2 Department of Radiation Oncology, Tongji University Affiliated Shanghai Pulmonary Hospital, Shanghai 200433, P.R. China

**Keywords:** stereotactic body radiotherapy, non-small cell lung cancer, radiation pneumonitis, respiratory motion management

## Abstract

Respiratory-induced tumor motion is a major obstacle in the precise delivery of stereotactic body radiotherapy (SBRT) for lung cancer, often leading to geometric uncertainties, insufficient tumor coverage and increased radiation-induced toxicity such as pneumonitis, esophagitis and rib fractures. The present review systematically assesses motion management techniques used in lung SBRT, synthesizing evidence from 352 high-quality clinical studies published between 2000 and 2024. Selected studies included patients with non-small cell lung cancer treated exclusively with SBRT which incorporated strategies such as deep inspiration breath-hold, abdominal compression, respiratory gating and real-time tumor tracking, and often integrated with image guidance technologies such as 4DCT, cone beam CT and MRI. These techniques demonstrated notable reductions in planning target volume margins and normal tissue dose, leading to improved local control and lower toxicity rates, particularly in tumors with large motion amplitudes or proximity to critical structures. Despite these benefits, implementation remains variable due to patient-specific challenges, technical complexity and institutional resource differences. The present review highlights the clinical applications and limitations of each strategy, and proposes a decision-making framework to guide clinicians in selecting the most appropriate motion management strategy based on tumor characteristics, motion amplitude and patient-specific factors. The integration of respiratory motion management with advanced imaging is essential for optimizing therapeutic outcomes and safety in lung SBRT.

## 1. Introduction

Lung cancer remains the leading cause of cancer-related mortality globally, with non-small cell lung cancer (NSCLC) accounting for ~85% of all lung cancer cases. Among patients with NSCLC, ~15% are diagnosed at an early stage, whilst those with locally advanced disease constitute 33–50% of the total patient population (1). Surgery is the standard treatment for stage I and II NSCLC; however, for patients who are ineligible for or refuse surgery, stereotactic body radiation therapy (SBRT) serves as an effective alternative (1–3). For locally advanced patients with NSCLC, SBRT in combination with surgery or other treatment modalities has emerged as a highly effective and low-toxicity strategy, demonstrating the potential to improve local control rates and offering an acceptable safety profile (4–6). Research indicates that the 3-year local control rate for SBRT in early-stage NSCLC can reach 90%, with long-term survival rates ranging from 60–80%, comparable to the outcomes achieved through surgical intervention (2,3). These promising results have led to increased interest and research into the use of SBRT for locally advanced patients with NSCLC (4,5).

Despite these clinical advancements, the success of SBRT hinges on two interdependent processes: Precise tumor delineation and the accurate delivery of radiation, which form the foundation for delivering high-dose, hypo-fractionated radiotherapy with curative intent in early-stage NSCLC (7). However, respiratory motion fundamentally compromises both aspects (8–11). Lung tumors may shift up to ≤3 cm during the respiratory cycle, causing substantial geometric mismatches between planned and delivered doses (12,13). These mismatches compromise tumor coverage and increase the risk of irradiation to adjacent normal tissues, notably leading to toxicities such as radiation pneumonitis (RP) (14–16), esophagitis (17), chest wall pain and rib fractures (18). The relationship between irradiated lung volume, mean lung dose and RP risk has been firmly established (14,19), making motion management a critical safety precaution in lung SBRT. Respiration-induced interplay between the moving tumor and the static beam also produces a dose-blurring phenomenon: The high-gradient SBRT dose cloud is smeared along the motion trajectory, effectively enlarging the volume of normal lung that receives intermediate doses, which widens the normal-lung V20 and mean lung dose, increasing the risk of RP (7).

To mitigate the impact of respiratory motion on SBRT for lung cancer, a wide range of respiratory motion management techniques have been developed, aiming to either account for or actively control tumor motion (20,21). Historically, motion-encompassing methods, such as internal target volume (ITV) expansion based on 4DCT, has been the default solution (22–24). Whilst simple to implement, this method often necessitates large margins, increasing dose to healthy tissues. By contrast, more advanced approaches, such as deep inspiration breath-hold (DIBH) (25), abdominal compression (AC) (26), respiratory gating (27) and real-time tumor tracking (28) attempt to mitigate or eliminate tumor motion altogether. These techniques are often integrated with image-guided radiotherapy (IGRT) modalities, such as cone-beam CT (CBCT) (29), 4D-CBCT (22) or MRI-guided systems (30), to monitor anatomy dynamically, verify positioning and ensure intra- and/or inter-fractional accuracy. When used effectively, the synergy between motion control and image guidance allows clinicians to shrink the planning target volume (PTV) margins, reduce radiation exposure to organs-at-risk (OARs) and decrease the incidence of RP (21) and other high-grade toxicities, particularly in patients with compromised pulmonary reserve or tumors near critical structures (31).

However, despite notable technological progress, the optimal selection and implementation of motion management techniques remain unresolved in clinical practice (32). Previous reviews have systematically summarized the fundamental principles and clinical applications of respiratory motion management techniques in lung SBRT (31,33–35), yet day-to-day clinical decisions remain driven by institutional habit and equipment availability rather than by stratified evidence. Variations in tumor characteristics (such as location, size and central-vs.-peripheral position) along with motion amplitude, collectively determine the dosimetric benefit and practical feasibility of each motion-management option; however, the field still lacks a consensus framework for integrating these variables into a unified respiratory-motion strategy (33). Central and ultracentral lesions, particularly those tumors abutting to critical structures such as the proximal bronchial tree, esophagus, major vessels or heart, exemplify this dilemma: Margins cannot simply be expanded, yet overly complex solutions may be unsustainable if a patient cannot maintain regular breathing or prolonged immobilization (31). The added treatment time and workflow complexity of advanced control strategies further discourage their routine use when clear, patient-level benefit has not been demonstrated. Consequently, a rigorous re-evaluation of both established methods and emerging technologies, framed around objective patient-selection and risk-stratification principles, is urgently needed to move from technology-driven to evidence-driven practice.

In light of these considerations, the present review aimed to comprehensively evaluate and consolidate the current understanding of motion management strategies in lung SBRT. Drawing upon data from 352 high-quality studies spanning from 2000 to 2024, the technical principles, clinical applications and toxicity outcomes of key motion management techniques are categorized and compared. Special attention is given to the interplay between imaging technologies and motion control strategies, with a focus on their effectiveness in reducing dose to normal tissues and improving local tumor control. Furthermore, the present review proposes a decision-making framework to guide clinicians in selecting the most appropriate motion management strategy based on tumor characteristics and motion amplitude. The present review also briefly introduces the clinical applications of new solutions, from artificial intelligence (AI)-driven motion compensation algorithms to MRI-guided adaptive radiotherapy and advanced surface monitoring systems, which are solutions that are expected to further enhance the precision treatment of lung SBRT.

## 2. Survey methodology

A comprehensive literature search was performed to identify studies focusing on the application of respiratory motion management techniques in SBRT for NSCLC. Databases such as PubMed (https://pubmed.ncbi.nlm.nih.gov/), Scopus (https://www.scopus.com/) and Web of Science (https://www.webofscience.com/) were searched using keywords including ‘SBRT’, ‘local control’, ‘radiation pneumonitis’, ‘respiratory motion management’, ‘motion control technologies’, ‘tumor tracking’, ‘breath-hold’, ‘respiratory gating’, ‘abdominal compression’ and ‘image-guided radiotherapy’ using logical OR and AND connectors. The search was limited to peer-reviewed articles published in English from January 2000 to December 2024.

The initial search yielded a total of 497 relevant articles. Studies were selected based on predefined inclusion criteria, which required that the study population consisted of adult patients with lung cancer aged ≥18 years, that the study involved motion management techniques in lung SBRT, and that it reported outcomes related to local tumor control and/or treatment-related toxicity. Studies with a follow-up duration of ≥6 months were included, along with randomized controlled trials, prospective/retrospective cohort studies and systematic reviews/meta-analyses. To ensure ‘high quality’, each study had to include a clearly documented motion management strategy (e.g., respiratory gating, breath-holding or tracking), complete outcome reporting (graded toxicities with incidence rates), use of image guidance (e.g., 3D CBCT, 4D-CBCT or online imaging), and the exclusive use of SBRT techniques (no conventional radiotherapy). Only studies involving human clinical subjects were considered. Following these criteria, 145 papers were excluded and a total of 352 studies were included for analysis. Furthermore, the literature cited in the present review were selected based on the thorough evaluation of these relevant studies.

## 3. Current status and challenges of respiratory motion management techniques

### Overview of respiratory motion management techniques

Respiratory motion management in lung cancer radiotherapy aims to minimize radiation exposure to healthy tissues, optimize tumor dose coverage and improve treatment accuracy. Since the 1990s, the development of SBRT has highlighted the impact of respiratory motion on treatment precision, leading to the development of several strategies, including image-guided methods (34), respiratory control techniques and motion compensation methods (35). Image-guided techniques monitor tumor position before or during treatment through imaging technologies, allowing for precise radiation beam delivery, such as in motion-compensating approaches that use 4DCT to assess motion and delineate internal target volumes. Respiratory control techniques aim to minimize lung and diaphragm motion by controlling the breathing of the patient, with methods such as AC (26) and breath-holding (25) commonly employed. Motion compensation techniques adjust radiation beam positions in real time during treatment to account for tumor motion, including techniques such as respiratory gating and real-time tumor tracking (27,35,36). Respiratory control and motion compensation techniques are often grouped together as respiratory motion management strategies (27,35). The combined use of image-guided techniques and respiratory motion management strategies enhances treatment accuracy, leading to improved therapeutic outcomes (31). The present section provides a short overview of these techniques, highlighting their principles, technologies and the challenges they face.

### Image-guided techniques

Since the development of lung SBRT in the 1990s, IGRT has undergone a notable evolution, transforming the paradigm of treatment accuracy and safety (27,37–40). In the early years, patient setup relied on rigid body frames and bony anatomy alignment using 2D portal imaging, which often resulted in substantial geometric uncertainties and necessitated large planning margins to account for setup and motion errors (37). The mid-2000s marked a pivotal shift with the integration of CBCT (39) and orthogonal kV imaging (40) into linear accelerators, enabling direct soft-tissue visualization and daily volumetric alignment, which markedly improved targeting precision and facilitated frameless SBRT delivery. Shortly thereafter, 4D-CT revolutionized motion assessment by capturing tumor trajectories throughout the respiratory cycle, laying the groundwork for individualized ITVs, respiratory gating and breath-hold strategies. Over the past decade, the field has advanced rapidly with the development of 4D-CBCT (22), surface-guided radiotherapy (SGRT) (41), real-time tumor tracking systems (42) and MRI-guided radiotherapy (MRgRT) (43), which offer unparalleled capabilities for real-time visualization, adaptive planning and motion-compensated dose delivery. In summary, advances in IGRT technology have made IGRT an essential component of lung SBRT, enabling high-dose, hypofractionated treatments to be delivered with sub-millimeter precision and minimal toxicity. Table SI (22,26,29,37–48) provides a comprehensive overview of the major IGRT modalities in lung SBRT, highlighting their technique principles, clinical applications, advantages, disadvantages and contributions associated with each approach.

### Respiratory motion management techniques

**Figure 1.**
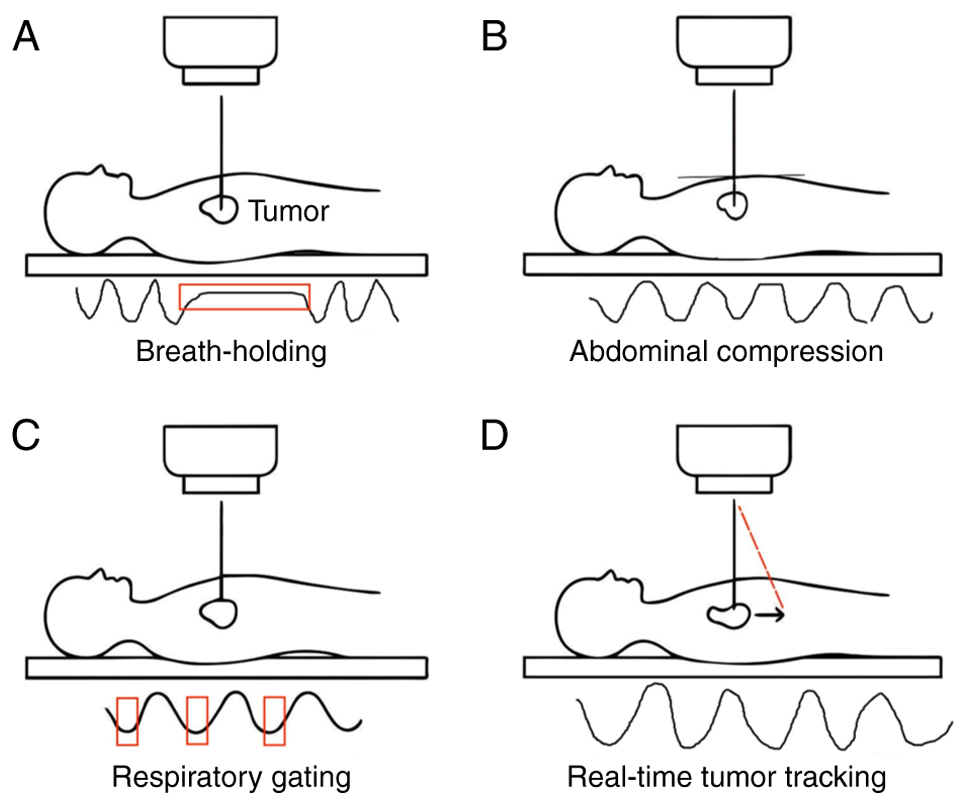
Schematic illustration of four respiratory motion management strategies in lung stereotactic body radiotherapy. (A) Breath-holding (deep inspiration breath-hold): Patient holds a deep inspiration to stabilize tumor position by expanding the lungs and lowering the diaphragm. (B) Abdominal compression: External pressure limits diaphragmatic movement to reduce tumor motion. (C) Respiratory gating: Radiation is delivered only during selected phases of the breathing cycle, guided by internal or external surrogates. (D) Real-time tumor tracking: Tumor position is continuously monitored and the beam dynamically follows the moving target.

According to the AAPM TG76 report, effective management of respiratory motion is critical in lung tumor radiotherapy, and respiratory motion management is necessary when tumor motion is >5 mm, as it can compromise imaging and dose delivery (35). Standard techniques for respiratory motion management (Fig. 1) include the following: i) DIBH, in which the patient voluntarily holds a deep inhalation during beam delivery to ‘freeze’ the tumor by expanding the lungs and lowering the diaphragm, thereby creating a stable target position (25); ii) AC, which applies gentle external pressure to the upper abdomen to mechanically limit diaphragmatic excursion and reduce the cranio-caudal range of tumor motion (26); iii) respiratory gating, where an external surrogate or internal fiducial signal is used to turn the beam on only during predefined phases of the breathing cycle, thus irradiating the tumor when its motion is minimal (32); and iv) real-time tumor tracking, in which the position of the tumor is continuously localized via implanted markers or image-based algorithms, and the beam or multileaf collimator dynamically follows the moving target throughout respiration (43). These methods aim to minimize motion-induced errors, thereby enhancing treatment precision and safety (27,35). Table SII (25,49–64) provides a comparative overview of these techniques, detailing their principles, clinical applications, advantages, disadvantages and contributions.

## 4. Clinical application and impact of motion management techniques in lung SBRT

### Clinical application of motion management techniques in lung SBRT

Accurate delineation of the tumor target volume, accounting for respiratory-induced tumor motion, is critical in lung SBRT. According to the International Commission on Radiological Units and Measurements Report No. 62 (65), the ITV encompasses the range of tumor positional variations throughout the respiratory cycle. Clinically, several respiratory motion management techniques are combined with imaging modalities to adaptively delineate the target volume based on individual tumor characteristics and respiratory dynamics (33). When the respiratory motion amplitude of the tumor is <5 mm, the commonly used approach is the motion-encompassing technique (35). This involves delineating the tumor target volume at each respiratory phase on 4DCT, then overlaying the tumor target volumes from 10 or 20 respiratory phases on the maximum intensity projection image to derive the ITV. A margin is subsequently added outside the ITV, taking into account factors such as positioning errors and anatomical variations, to define the PTV. However, due to the complexity and unpredictability of lung respiratory motion, the ITV may be influenced by factors such as the respiratory cycle, amplitude and frequency, leading to uncertainties in its delineation (35).

When the amplitude of tumor respiratory motion is >5 mm, it is essential to create an individualized radiotherapy plan, and appropriate respiratory motion control techniques should be selected based on the specific condition of the patient to accurately define the ITV (35). With breath-holding techniques, the internal target volume (ITV), which now equals to gross target volume (GTV), is typically delineated on the 3D images from specific respiratory phases (end of inspiration or end of expiration) of the 4DCT dataset (66,67). For AC techniques, the target volume is usually delineated during the deep inhalation phase of the 4DCT scan (68), which markedly reduces the influence of respiratory motion on the target volume. In respiratory gating, the respiratory phases within the preset gating window are averaged to form an average density projection from the corresponding subset of the 4DCT data, which captures residual motion within the gating window. In this case, the ITV is derived from the blurred target volume in the subset of 4DCT images (69). In real-time tumor tracking techniques, target volume delineation is typically achieved using the ITV method, where the tumor is delineated across selected respiratory phases and these volumes are combined to form an ITV (70). Compared with other respiratory motion management methods, real-time tumor tracking excels in tracking and compensating for tumor motion, thereby reducing target volume displacement and minimizing radiation exposure to normal tissues (71).

During the implementation phase of lung cancer SBRT, respiratory motion management serves a critical role. In practice, IGRT techniques facilitate real-time monitoring and assessment of both the respiratory motion of the patient and the tumor position. This data allows clinicians to adjust the treatment plan for more precise tumor localization, reducing radiation damage to surrounding normal tissues (21). Combining IGRT with several respiratory motion control techniques, such as breath-holding, AC, respiratory gating and real-time tumor tracking, helps to reduce the uncertainty caused by lung motion, thereby improving both treatment precision and safety (28). Due to the high doses and extended treatment duration involved in lung SBRT, not all respiratory motion management techniques are suitable for this form of therapy. Therefore, a comprehensive evaluation of the condition and treatment requirements of the patient is essential. Clinicians must assess factors such as tumor location, size, motion amplitude and respiratory stability to select the most appropriate technique (33,35).

### Respiratory motion management techniques and toxicity outcomes

The adoption of SBRT for lung tumors, whilst offering excellent local control, necessitates precise motion management to mitigate radiation-induced toxicities. Studies employing motion-encompassing, DIBH, AC, respiratory gating and real-time tumor tracking report acute grade 3 or higher toxicity rates that vary widely from 2.1–59% (18,31,72–81). For instance, one study reported a 10.1% incidence of grade 3+ pneumonitis with respiratory gating in patients whose tumors exhibited roughly 1 cm motion (74), whilst another study reported 12 high-grade toxic events in a cohort of 70 patients treated with real-time tracking (31). Late toxic effects also vary. In certain reports, chest wall pain was reported in 5–25% of patients (75) and rib fractures in 0–18% (76), whilst grade ≥3 late toxicity reached 14.6% in certain studies (18). These radiation-induced toxicities are notably influenced by how respiratory motion is accounted for (77,79). Furthermore, these outcomes depend heavily on how effectively respiratory motion is controlled and the degree to which image guidance improves target localization (21).

To identify the most suitable respiratory motion management technique for patients, the present section assesses the application of different respiratory motion management techniques in lung SBRT and compares their impacts on treatment outcomes and toxicities. Particular attention is paid to their interaction with image guidance modalities in both peripheral and central lung tumors.

### Motion-encompassing

The motion-encompassing method is the most widely adopted and operationally straightforward motion management technique. It employs 4DCT to contour the entire range of tumor motion during free breathing, thereby generating an ITV that is expanded to a PTV with additional setup margins (82). This technique is particularly useful for patients with moderate tumor motion (<5 mm in 3D) and regular breathing patterns who cannot tolerate more advanced motion management methods, such as breath-holding or respiratory gating (27,35). Whilst this approach ensures geometric coverage without the need for real-time monitoring, the trade-off is a volumetrically larger PTV that inevitably irradiates more normal tissue (83).

As the PTV is expanded, more normal lung and nearby tissues fall within high-dose regions compared with gating or tracking. For instance, a planning study reported that free-breathing (SBRT plans had markedly larger high-dose lung volumes than gated or tracked plans (82). In that comparison, the lung V20 (volume receiving 20 Gy) was highest with free-breathing (6.34% of ipsilateral lung), compared with 4.96% with gating and only 3.82% with real-time tracking. The larger ITV-based PTV improved nominal target coverage slightly, but at the cost of greater normal lung irradiation. Clinical data also reflect this: Patients treated without active motion management tend to have a higher mean lung dose and lung V20, known predictors of pneumonitis (84). As a precaution, strict lung dose constraints are required by clinical protocols (such as RTOG 0236, 0813 and 0915) (85–87). With such constraints, reported rates of grade ≥3 pneumonitis after peripheral SBRT are <10% (88). However, for central tumors, especially those near the proximal bronchial tree or mediastinum, motion-encompassing based SBRT poses a greater risk (89,90). Therefore, whilst motion-encompassing based ITV planning remains viable for peripheral tumors with limited motion, its use in central lesions is often constrained by the risk of overdosing OARs, unless fractionation is adjusted or margins are aggressively minimized (91).

### DIBH

DIBH is a technique where patients take a deep breath and hold it for a short duration during treatment, typically under audio-visual coaching (92). DIBH immobilizes the tumor and also alters thoracic geometry: The lungs expand, the diaphragm lowers and the mediastinal structures are pushed apart. DIBH can be performed with an active device (such as the Elekta Active Breathing Coordinator) that blocks breathing at a certain lung volume, or with voluntary breath-hold using coaching and monitoring (often with a visual feedback system or surface tracking to ensure reproducible inhale depth) (93,94). In lung SBRT, DIBH is less common than gating for motion management, but it has distinct advantages: It does not requires a continuous beam interruption (the beam is delivered during each held breath, typically multiple breath-hold cycles per fraction), and the deep inhale itself can increase lung volume, thereby reducing lung dose for the same absolute irradiated volume (95). This geometric advantage reduces the irradiated lung volume for lung tumors, thereby lowering lung Vx metrics and associated toxicity risks. Dosimetric comparisons between DIBH and free-breathing plans have revealed marked reductions in lung V5-V20, mean lung dose, mean heart dose and chest wall V30 (25,95,96). These improvements are particularly valuable in minimizing the risk of radiation pneumonitis and late cardiac events. For peripheral tumors near the chest wall, DIBH reduces the volume of irradiated bone and muscle, thereby lowering the incidence of chest wall pain and rib fractures (25). Moreover, for mid- and lower-lobe tumors located central tumors, DIBH is especially beneficial for these kinds of tumors. DIBH can reposition the tumor relative to the proximal bronchial tree and esophagus, potentially enabling more conformal avoidance of these OARs (95).

### AC

AC uses an external constraint (such as a compression plate or belt pressed against the upper abdomen) to restrict diaphragmatic excursion and thus reduce the range of lung motion (97). It is a ‘passive’ motion management approach, in which the patient breathes freely but with a smaller amplitude. This technique is particularly considered for patients with notable respiratory motion, especially those with lower lobe tumors due to their proximity to the diaphragm. During SBRT planning, patients are immobilized using devices such as the Stereotactic Body Frame or BodyFix system. A compression belt is placed 3–4 cm below the costal margin and tightened to a comfortable level that effectively reduces motion amplitude (98). 4DCT scans are often used to assess tumor motion with and without AC, guiding treatment planning and ensuring accurate target (26).

The evidence on toxicity is limited, but studies suggest AC may lead to worse local control in certain cases, potentially due to increased interfractional variability, which could indirectly affect toxicity if it results in under-treatment (41). A retrospective analysis of 47 patients with NSCLC and tumor motion ≥8 mm reported no significant differences in overall survival (OS) or disease-free survival (DFS), with 3-year OS rates of 54.4% with AC vs. 52.4% without (P=0.909), and DFS rates of 34 vs. 38.1%, respectively (P=0.639) (98). However, stratified analysis reported lower local control for patients with an unfavorable prognosis (RPA Class II), with local control rates of 50.5% with AC vs. 80.0% without (P=0.394), suggesting potential indirect toxicity risks. Direct toxicity data specific to this technique are scarce, but its impact on variability may contribute to increased normal tissue exposure. Overall, when properly implemented as part of a comprehensive image-guided SBRT protocol, AC appears to enhance the therapeutic ratio by limiting collateral dose and mitigating toxicity in lung cancer treatment.

### Respiratory gating

In respiratory gating, the radiation beam is turned on only during a specific portion of the breathing cycle when the tumor is in a favorable, predictable position (32). A gating window (for example, 30–40% of the breathing cycle) is defined; during treatment, the patient's breathing is monitored (via external surrogates such as an abdominal marker block or internal fiducial tracking) and the linear accelerator automatically triggers the beam on when the cycle enters the chosen window and off as the tumor leaves (56). This effectively ‘freezes’ tumor motion during irradiation, allowing a smaller margin (32). 4DCT simulation is used to choose the window (for example including only phases around end-exhale or end-inhale) and to delineate a gated ITV (32). This technique allows for reduced internal margins, leading to decreased PTV volumes and sparing of normal tissues (99).

Gating can markedly lower normal tissue dose by restricting radiation to optimal tumor positions, reducing margins and motion blurring. A planning study reported that gating around exhalation (20% duty cycle) decreased mean lung dose (~0.6 Gy), lung V20 (~2.4%), esophagus D5cc (2 Gy) and maximum heart dose (>3 Gy), with modeled normal tissue complication probability for pneumonitis decreasing from 11 to 7% (56). Clinically, gated SBRT yields local control and toxicity outcomes comparable with ITV-based plans, with retrospective data reporting no increase in high-grade toxicity (100). Gating is especially valuable for central tumors or dose-limiting OARs, enabling safer escalation by improving dose conformity (56). Though gating may prolong treatment and depend on regular respiration, advances in monitoring and delivery have enhanced efficiency, making it feasible in appropriately selected patients.

### Real-time tumor tracking

Real-time tumor tracking represents an advanced motion management strategy wherein the radiation beam continuously follows the spatial trajectory of the tumor throughout the respiratory cycle, obviating the need for phase-specific beam gating (101). Implementation requires accurate, real-time localization of the tumor via fiducial markers (detected by kV imaging or electromagnetic transponders), direct tumor visualization (such as cine-MRI), or correlation models linking external surrogates with internal motion (102–104). Systems such as CyberKnife employ stereoscopic imaging and robotic arm corrections to dynamically align the beam (105), whilst Vero and select C-arm linacs achieve similar functionality through real-time gantry or multileaf collimator (MLC) adjustments (48,106). MLC tracking enables beam adaptation by modulating the aperture in synchrony with target motion (107). Treatment planning typically utilizes 4DCT to characterize respiratory motion, but instead of generating a composite ITV, plans are formulated on a representative phase or an average CT dataset, permitting substantially reduced PTV margins, often as low as 2–3 mm, to account only for system latency and localization uncertainty (108,109). The elimination of large motion margins markedly limits normal tissue exposure and enhances spatial dose conformity (108).

Among all motion mitigation strategies, real-time tracking achieves the most pronounced reduction in irradiated normal tissue volumes by minimizing both geometric expansion and temporal blurring of the dose distribution. Dosimetric analyses have consistently demonstrated that tracking outperforms gating and ITV-based methods in lung sparing; for instance, Prunaretty *et al* (82) reported a reduction in ipsilateral lung V20 from ~6.3% with ITV planning to ~3.8% with tracking. Moreover, PTV volumes have been reported to decrease by >60% in certain cases, with MRI-guided tracking studies achieving median PTVs of ~16.5 cc compared with ~43.6 cc for comparable ITV-based approaches (110). These reductions translate into enhanced sparing of adjacent critical structures such as the lung, esophagus and heart, and potentially lower risks of toxicity such as radiation pneumonitis. Clinical series, particularly those employing fiducial-based robotic tracking, report notable local control rates (such as 100% at 2 years) with a low incidence of grade ≥2 toxicity (111). Whilst real-time tracking offers superior tumor targeting, it presents unique challenges, notably the risk of unintended OAR exposure due to uncorrected tumor-OAR displacement throughout the breathing cycle (112). Furthermore, continuous imaging slightly increases treatment time and imaging dose; however, these are generally well-controlled with modern low-dose protocols and safety mechanisms (113). Despite these considerations, tracking remains a highly effective modality for enhancing dose precision and reducing toxicity in lung SBRT (114).

### Patient-specific motion management decision for lung SBRT

Each technique has its unique approach to handling respiratory motion, and their toxicity profiles vary based on mechanisms and patient populations (115). The choice of technique should be individualized based on patient characteristics, tumor location and ability to cooperate (112,116). For example, patients with notable respiratory motion and lower lobe tumors may benefit from real-time tumor tracking or gating (112), whilst those with moderate motion or the inability to perform breath-hold may be more suited for motion-encompassing approaches (22–24). Daily IGRT is essential to reduce setup uncertainties and ensure accurate delivery, especially when larger margins are used (117). Table SIII summarizes the clinical applications of motion management techniques in lung SBRT to guide method selection.

## 5. Challenges and future prospects of respiratory motion management in lung SBRT

### Overcoming barriers in respiratory motion management

SBRT for early-stage lung tumors achieves high local control with ablative doses, but respiratory motion remains a critical obstacle to precision (118). Effective motion management is essential for maintaining tight treatment margins and sparing normal tissue, yet current approaches are challenged by not only clinical variability (119) but also technological limitations (56). Emerging technologies promise to address these issues and pave the way for adaptive, personalized treatments (120–125).

### Clinical challenges in motion management

#### Patient compliance and variability

SBRT delivery often relies on techniques such as breath-hold or coached breathing, but not all patients can reproducibly comply. Whilst DIBH has shown high compliance in numerous patients with lung cancer, certain patients (such as those with poor pulmonary function or anxiety) struggle to maintain consistent holds (25). Even with coaching, breathing patterns can vary day to day, and baseline drift of the respiratory cycle can occur during treatment. In one study using real-time tumor tracking, ~42% of lung SBRT cases exhibited a cumulative tumor baseline shift of >3 mm within 10 min of beam-on (126). This intrafraction drift toward a new breathing baseline underscores the need for continuous monitoring and adaptation to prevent target miss or overdose. Additionally, patient comfort measures (immobilization frames and AC) can be uncomfortable, and coughing or involuntary motion presents unpredictable challenges (127,128).

### Anatomical changes and tumor location

Although lung SBRT is delivered in a short course, anatomical changes between fractions are possible, for example, tumor shrinkage, pleural effusions or atelectasis resolution can alter internal geometry (25). Interfraction changes are usually modest but can affect daily alignment in high-precision treatments (129). Tumor location also influences the motion management strategy (21,112,116). Peripheral lung tumors near the diaphragm may have larger motion amplitudes but are farther from critical central structures (130). Central or ultra-central tumors (adjacent to major airways or vessels) pose a different challenge: Their motion is often less (due to anchoring in mediastinum) but even small deviations can risk high-dose exposure to sensitive organs (131). SBRT for central lesions is frequently fractionated into 5–8 sessions (compared with 3–5 sessions for peripheral lesions) to mitigate toxicity (132,133). Despite caution, toxicity limits remain a concern, for example, high-dose SBRT near bronchi can cause severe toxicity such as airway necrosis or hemorrhage (132). A phase I/II trial (RTOG 0813) assessing 5-fraction SBRT for central tumors reported that the maximum tolerated dose was 12 Gy ×5, achieving 88–89% 2-year local control rates with acceptable toxicity (86). However, more aggressive dosing in ultra-central locations has led to fatal pulmonary hemorrhage in a notable subset of patients (132). These outcomes highlight the delicate balance between tumor control and toxicity: Motion management must be coupled with judicious dose constraints, especially centrally.

### Technological limitations in motion management

#### Gating and tracking system limitations

Current respiratory gating systems typically use external surrogates (infrared markers or belts) to time radiation delivery with specific breathing phases. A fundamental limitation is the imperfect association between external signals and internal tumor motion. Even with careful calibration, residual uncertainties persist: Tumors can move out of the gated window despite a stable external signal, especially if the relationship drifts over time (134). Even with markers, tracking involves extra imaging (orthogonal X-rays or continuous kV fluoroscopy) which adds imaging dose and potential motion blur. Markerless tumor tracking using onboard imaging is under active development; however, reliably distinguishing the tumor on X-ray images without high-contrast markers is challenging, especially for small lesions overlapping bony structures (135,136). Recent advances in real-time image processing are beginning to address this, but widespread clinical implementation is pending further validation.

### Imaging and planning uncertainties

Another technological hurdle lies in accurately quantifying motion during simulation and translating that into the treatment plan. 4DCT is the workhorse for motion assessment, generating phase-resolved images to delineate ITV. However, 4DCT has well-documented uncertainties: It provides an average motion envelope but often underestimates the true extremes of motion (27). Irregular breathing or slow gantry rotation can produce artifacts (such as incomplete tumor trajectories and duplicate phase images) that misrepresent tumor motion. Studies comparing 4DCT-measured motion with actual tumor motion during treatment have reported that 4DCT captures the mean motion well but can miss peak excursions by ~2 mm on average (27,137,138). Consequently, an ITV based on 4DCT may be too small if the patient exhibits more pronounced motion on the treatment day. By contrast, using generous margins to cover this uncertainty erodes the benefit of motion management. Imaging quality is another constraint: Standard CBCT for setup verification blurs moving anatomy by averaging over the respiratory cycle (139). New 4D-CBCT methods can capture tumor motion on the day of treatment, improving visualization of the trajectory of the tumor and enabling tighter adaptive margins (140). However, 4D-CBCT involves longer scan times and a higher imaging dose, and its integration into routine clinical workflow is still evolving (140). For centers without 4D-CBCT, verifying tumor position in a specific breathing phase remains difficult, leading several to rely on surrogates or slower repeated 2D imaging during treatment (22).

### DIBH and AC challenges

DIBH and AC in lung SBRT have been reported to markedly shrink the target volume and reduce doses to the heart, lungs and chest wall (25). DIBH demands that patients repeatedly hold their breath for 20–30 sec, a task that proves difficult for individuals with impaired lung function, often resulting in inconsistent breath-holds and potential shifts in tumor position. Similarly, AC, whilst useful for managing motion in lower lobe tumors, offers limited benefits for upper or middle lobe tumors and may cause discomfort, potentially disrupting breathing patterns. Modern surface-guided systems provide visual feedback and gating to ensure the beam fires only when the breath-hold is within a tight threshold (134). Nevertheless, if a patient's performance degrades over a fraction (due to fatigue or discomfort), treatment must be paused or aborted to maintain accuracy (98). Consequently, whilst these methods can be highly effective, their success hinges on the lung capacity of the patient and their tolerance for discomfort, rendering them less universally applicable across all patients (141).

### Emerging technologies and solutions

Despite the challenges, recent developments are paving the way to more effective and accessible motion management in lung SBRT. The present section highlights promising current and emerging technologies aimed at overcoming the limitations.

### MRgRT and on-line adaptation

Integrated MRI-linear accelerators, such as the Unity MR-Linac by Elekta and MRIdian by ViewRay, now provide real-time soft-tissue visualization, enabling adaptive gating or tracking based on the actual location of the tumor (120,142). This approach not only compensates for interfraction anatomical changes but also allows for online adaptive planning tailored to each patient's daily anatomy. For example, a study on lung SBRT using MRI-Linac reported a 12-month local control rate of 95.6% with low toxicity, underscoring its clinical efficacy (143). Additionally, ongoing phase III clinical trials, such as the MIRAGE trial, evaluating MRI-guided SBRT for prostate cancer, demonstrate the potential for broader clinical adoption and translation (144).

### Surface tracking and markerless monitoring

Non-invasive SGRT offers continuous monitoring of patient motion without additional radiation. When combined with advanced AI-driven markerless X-ray tracking, these systems may provide a comprehensive, real-time picture of both external and internal tumor positions (121,122). Moreover, these advancements in markerless tracking have shown promise in reducing setup errors in lung SBRT (122).

### AI-based motion prediction and adaptive workflows

AI is emerging as a powerful tool to predict respiratory motion and drive dynamic treatment adaptations (123,145). AI algorithms, such as artificial neural networks, can forecast the future position of the tumor to overcome system latencies, achieving root mean square errors of 0.5–0.9 mm within prediction windows of 120–520 millisecond (124). These algorithms adjust MLC settings and support closed-loop feedback systems that automatically compensate for motion-induced dose errors (107,125,146). Ongoing research aims to integrate these AI models into clinical workflows, enhancing real-time adaptation in SBRT (147).

### Multi-modality imaging integration

The fusion of imaging modalities, such as 4D-PET/CT, 4D-MRI and ultrasound, can improve tumor localization and delineate motion patterns more accurately (115,148). Deformable image registration between these modalities is key to creating a consistent anatomical framework, enabling more precise motion-adaptive treatments (149). These novel techniques are currently under clinical research.

## 6. Conclusion

The present review comprehensively discussed the principles and technologies behind several respiratory motion management strategies, including motion-encompassing, DIBH, AC, respiratory gating and real-time tumor tracking. These techniques, when appropriately applied, have been shown to be effective in reducing treatment-related toxicities, whilst also improving local control in patients with NSCLC. However, despite these advancements, challenges remain, including variability in patient compliance, tumor location and motion amplitude, which complicate the optimal selection and implementation of these methods.

Future research should focus on optimizing these techniques for more precise and personalized clinical applications, particularly for patients with central or ultracentral tumors who are at higher risk for severe toxicity. Additionally, long-term clinical studies and follow-up data are essential to improve the understanding of the efficacy of respiratory motion management in reducing both acute and late toxicities, as well as improving overall treatment outcomes. Innovations in imaging technologies, such as MRI-guided radiotherapy and AI-driven motion compensation, hold promise for enhancing the precision of motion management and reducing radiation exposure to healthy tissues. Ultimately, the continued development of novel motion management techniques and their integration with advanced imaging modalities will contribute to safer, more effective SBRT treatments for patients with lung cancer, improving both local control and quality of life.

## Supplementary Material

Supporting Data

## Data Availability

Not applicable.
