# Supplementary material for: Application of respiratory motion management technology for patients with lung cancer treated with stereotactic body radiotherapy (Review)
Source: Oncol Lett. 2025 Jun 27;30(3):415. doi: 10.3892/ol.2025.15161 (PMC12235356; doi:10.3892/ol.2025.15161)
Supplement: Supporting Data [file Supplementary_Data.pdf]

Table SI. Classification and characteristics of imaging guidance equipment.

Category	Imaging principle	Clinical applications	Advantages	Disadvantages	Contributions
Electronic portal imaging device	Utilizes MV energy for position verification via electronic portal imaging (such as Varian TrueBeam)	Position verification, field verification and real-time dose monitoring (37)	Integrated on every linac and fast	High imaging dose and susceptible to interference from metallic objects	Serves as a basic positional check (37)
CBCT	Utilizes kV or MV X-rays to acquire high-resolution 3D images through CBCT (such as Varian TrueBeam and Elekta Synergy)	Daily setup verification and intrafraction monitoring (39)	Fast imaging speed, high resolution and low radiation exposure	Requires regular quality assurance and accurate alignment of imaging and treatment beams required	Allows identification of anatomical changes over treatment; and shrinks residual setup error to ~2 mm, allowing PTV margin reduction of 30-50% (44,45)

4D CBCT	Uses time-resolved CBCT imaging to capture multi-phase images throughout the respiratory cycle (such as Varian TrueBeam)	Daily setup verification and intrafraction monitoring, and used for respiratory motion management (22)	Can track tumor motion throughout the entire respiratory cycle and improves localization accuracy	Higher imaging dose and requires additional hardware and software support	Enhances accuracy for mobile tumors by reducing motion blur; cuts registration uncertainty by a further 1.4 mm; and permits individual ITV reductions of 25-35% for tumors with ≥1 cm motion (22,26,46)
FBCT	Employs narrow-beam X-rays for thin-slice scanning to minimize scatter effects (such as uRT-linac 506C)	Provides precise electron density values for dose calculations (47)	High resolution, low artifact sensitivity and improves dose	Higher radiation exposure and costly equipment	Improves dose-calculation accuracy to within ±1% (47)

			calculation accuracy		
kV X-ray stereoscopic Imaging	Utilizes X-ray images from multiple angles to generate 3D images (such as CyberKnife)	Continuous/periodic intrafraction target tracking (40)	Provides 3D images and enhances depth visualization of the tumor	Requires either visible tumor or implanted markers; high cost; and increases radiation exposure	Key to real-time tumor tracking in CyberKnife, and enables margins as small as 2-3 mm with local control ~95% (48,49)
Fluoroscopy imaging	Utilizes 2D X-ray (kV/MV) to generate real-time dynamic images (such as Varian TrueBeam)	Real-time guidance for tumor localization and treatment monitoring (38)	Direct observation of motion and integrated with gating	Lack of depth information; ambiguous if the tutor overlaps other structures in projection; and high radiation dose	Aids in verifying motion during gating and tracking, and improves intrafraction accuracy, reducing geographic miss (38)

Optical surface monitoring system	Utilizes optical cameras to create 3D surface map, detecting sub-mm shifts and respiratory motion (such as AlignRT)	Real-time monitoring of patient posture and respiratory motion during radiotherapy(41)	No additional radiation exposure, easy to use and compatible with multiple systems	Provides only surface data, and sensitive to lighting and environmental conditions	Reduces interfractional set-up error with mean values of <5 mm and <0.5° in all directions(41)
Electromagnetic navigation tracking system	Percutaneous/bronchoscopic gold marker implantation, tracked via X-ray/EM signals (such as Calypso)	Real-time tumor position tracking (42)	No imaging dose and true 3D internal target	Invasive implantation, risk of migration and expensive	Offers sub-mm real-time tracking, and improves tumor control with low toxicity (42)
MRI-guided Systems	Integrates MRI with linear accelerators to provide real-time high-quality soft tissue imaging during treatment (such as Elekta Unity)	Daily on-line adaptation and real-time soft-tissue gating (43)	Excellent soft tissue contrast, adaptive planning and no ionizing	Complex equipment, high cost, long session times and unsuitable for patients with pacemakers or metallic	Real-time tumor gating and daily adaptive planning improve dose coverage and safety,

			radiation from imaging	implants due to potential motion artifacts and magnetic field distortions	and 1-year LC ~95% with low Grade3+ toxicity (43)

MV, megavoltage; CBCT, cone beam CT; PTV, planning target volume; ITV, internal target volume; FBCT, Fan Beam CT; LC, local control.

Table SII. Classification and characteristics of respiratory motion control techniques.

Techniques	Technique principle	Clinical applications	Advantages	Disadvantages	Contributions
Breath-holding	The patient inhales deeply or exhales fully and holds their breath (such as Active Breathing Coordinator)	Used in diagnostic imaging (such as CT scans) and short-term radiotherapy to reduce motion artifacts (25,50–52)	Reduces motion artifacts, enhances image quality and improves target delineation	Requires a high degree of patient cooperation, and certain patients may find it challenging to tolerate	Residual motion ≈0 mm during hold; allows PTV margins of 2-3 mm; and reduces mean lung dose >20% (50–52)
Abdominal compression	Reduces diaphragm motion through abdominal pressure (such as Body Pro-Lok ONE™ Respiratory	Used for radiotherapy of abdominal or thoracic tumors to stabilize diaphragm motion (53–55)	Simple to implement, low equipment requirements and markedly reduces diaphragm motion	Reduces patient comfort and its applicability is limited by the tolerance of the patient	Reduces motion amplitude by ~50% and permits PTV margin reduction from ~10 mm to ~6 mm (53–55)

	Belt and Bellyboard)				
Respiratory gating	External surrogate (infra-red marker block, spirometer or SGRT) triggers beam-on only during a pre-defined phase window (such as RPM/RGSC)	Used in precise radiotherapy for lung or liver cancers, and synchronized with respiratory cycle (56–60)	Synchronized with patient breathing, reducing the chance of target miss and improving treatment accuracy	Longer treatment time and less effective if the breathing is irregular	Residual motion <5 mm; PTV margin ~5 mm; and lung V20 reduced by 1-2 % vs. ITV plans (56–60)
Real-time tumor tracking	Tracks markers or tumor position and dynamically adjusts radiation based on real-time	Used in advanced radiotherapy for mobile tumors (such as lung cancer or pancreatic cancer)	High precision, reduces radiation exposure to normal tissues and improves	High technical demands and longer treatment time	Residual motion 2-3 mm and enables PTV margins of 2-3 mm (61–65)

	imaging (such as Synchrony and ExacTrac Dynamic)	and guided by real-time imaging (61–65)	consistency of dose delivery to the target		

PTV, planning target volume; ITV, internal target volume; SGRT, surface-guided radiotherapy; RPM, real-time positioning management; RGSC, respiratory gating for scanners.

Table SIII. Motion management decision table for lung SBRT based on tumor location, size and respiratory motion amplitude.

Location	Size	Motion amplitude (peak-to-peak)	Recommended motion control strategies	Image guidance modalities (planning and treatment)
Peripheral (away from mediastinum)	Small (≤3 cm)	Low (<5 mm)	ITV-based planning	4DCT for ITV generation (or breath-hold CTs for inhale/exhale); and daily kV CBCT for setup verification (using bony anatomy or tumor if visible)
		Moderate (5-10 mm)	Active motion management recommended: Such as abdominal compression or respiratory gating; if available, real-time tumor tracking; and DIBH is another option if patient can comply.	4DCT planning with compression device (if used) to assess residual motion; if gating: 4DCT to define gating window (such as 30-70% phase), external respiratory sensor (or internal fiducial) for real-time monitoring, and pre-treatment fluoroscopy to verify tumor motion within gating window; if tracking:

				Implant fiducial markers, use real-time kV X-ray imaging (such as CyberKnife); daily CBCT for setup (performed at a consistent breathing phase or with compression on); and if DIBH: Planning CT in breath-hold, surface tracking or spirometry for breath-hold reproducibility, and verify with gated CBCT/fluoroscopy at breath-hold.
		High (>10 mm)	Strong motion mitigation required: Combine methods if needed. Respiratory gating (tight gating window at end-exhale) or real-time tracking are first-line to keep motion <5 mm. Use abdominal compression concurrently to reduce baseline motion and increase gating	4DCT simulation to quantify large motion and design strategy. Possibly repeat 4DCT with compression to evaluate improvement; if gating: Narrow gating window (such as 30-50% phase) to minimize residual motion. Used external marker or internal fiducial for gating signal. Verify tumor position in gating window via fluoroscopy. Physicist/therapist monitors

			efficiency. If patient can do breath-hold, treat in repeated DIBH cycles as an alternative (complete motion elimination).	real-time trace during treatment; if tracking: Fiducials + continuous kV imaging throughout treatment (such as 30-60 sec interval imaging in robotic tracking); daily CBCT (or 4D-CBCT) for initial setup. Intrafraction monitoring via surface camera (for DIBH) or cine imaging (for gating/tracking).
Peripheral	Medium (3-5 mm)	Low (<5 mm)	ITV-based planning (4DCT-derived ITV) without gating. Ensure robust setup alignment given tumor size.	4DCT for ITV. Consider mid-ventilation planning as an alternative (average tumor position with margin); and daily CBCT for setup. Use tumor matching if visible, or anatomical surrogate if not.
		Moderate (5-10 mm)	Active management (similar to small tumor): Compression or Gating to shrink motion envelope; consider DIBH or tracking if	4DCT to delineate motion range. Test compression efficacy; gating setup with external surrogated and 4DCT phases if used. CBCT with gating or breath-hold technique for

			appropriate. Combination approaches (compression + gating) can be utilized.	alignment; and fiducial placement for tracking if standard IGRT is challenging (large tumors may obscure visualization).
		High (>10 mm)	**Aggressive motion control: Gating or tracking** strongly indicated (to avoid ≥1 cm extra margin). Use **compression** to assist. **DIBH** if feasible. If none of these are possible, consider treating with alternative fractionation (beyond SBRT scope).	Same as peripheral small/high-motion: Thorough 4DCT evaluation. Employ gating with verification, or tracking with fiducials; and use multiple image guidance tools: 4D-CBCT or repeated CBCT mid-treatment to ensure no shift, given large motion potential.
Peripheral	Large (>5 cm)	Low (<5 mm)	**ITV or gated** plan (depending on proximity to OARs). Large tumors have less relative benefit from gating if motion is minimal, but if near chest wall, one might gate to	4DCT for ITV; CBCT for setup. Consider **fiducials** if tumor has poor image contrast (some large tumors cause atelectasis making edges unclear on x-ray); and if gating chosen

			reduce chest wall dose. Generally, **ITV-based** approach with 4DCT is acceptable if <5 mm motion.	(such as to spare critical normal tissue), implement the aforementioned method.
		Moderate (5-10 mm)	Active management required: Prefer respiratory gating (to minimize margin for this large target) or DIBH (which also expands lungs, potentially reducing dose to lung/heart). Compression may be less effective for upper lobe tumors (common for large central tumors), but can be tried for a lower-lobe lesion. Tracking is generally not favored for very large tumors (due to complexity), but	4DCT to guide gating or breath-hold planning. Possibly plan on a gated mid-position CT or inhale/exhale breath-hold CTs.; use image guidance focusing on nearby OAR positions (such as ensure tumor clearance from mediastinal structures on gated CBCT); and monitor intrafraction with surface tracking (for DIBH) or fluoroscopy (for gating).

			could be attempted with multiple fiducials.	
		High (>10 mm)	**Maximum intervention: Gating and/or DIBH** nearly mandatory for safe treatment. Aim to treat in a phase or breath-hold where tumor motion is minimized. Consider partial **ITV + gating hybrid** (such as gating to cover most motion but accept a certain residual if needed with slightly enlarged ITV). **Tracking** with fiducials could be considered in extreme cases, but large tumor motion may also involve deformation. Multi-fraction (hyperfractionated SBRT) may be	Comprehensive 4D imaging and possibly fluoroscopic simulation to understand motion trajectory; strongly consider DIBH CT simulation and treatment, especially if the tumor is central (to reduce proximity to organs); gating: Use the smallest effective window and verify extensively that the tumor stays in field (such as intrafraction cine imaging); and employ all relevant IGRT: For example, soft-tissue matching on CBCT and fiducial-based alignment if markers present.

			considered to mitigate risk (per institutional protocol).	
Central (near hilum/mediastinum)	Small (≤3 cm)	Low (<5 mm)	**ITV-based planning** (4DCT) is acceptable **if** critical organs can be spared with normal margins. However, consider **gating or breath-hold** even for small motion if tumor is very close to OARs (to tighten the margin). Use careful image guidance (tumor may be less visible amid mediastinal structures).	4DCT for ITV; consider obtaining breath-hold CTs to evaluate tumor position relative to heart/mediastinum in inhale vs exhale; daily CBCT (soft-tissue window) for setup; **fiducial markers** may be placed in or near tumor to aid localization if the tumor is hard to visualize; and if gating/breath-hold used electively, follow those IGRT procedures.
		Moderate (5-10 mm)	**Respiratory gating** (preferred) or **DIBH** to restrict motion and shrink margins around tumor and adjacent OARs. **ITV alone is not ideal** due	4DCT to quantify motion and plan gated or breath-hold strategy. Possibly use mid-ventilation or gating-phase imaging for planning.; if gating, use an external respiratory

			to proximity of critical structures: Active management is recommended to keep the high-dose region tight. Abdominal compression may be of limited benefit unless the tumor is lower-central, but it can be attempted.	signal and verify tumor-OAR geometry in gating window via imaging (such as tumor distance from carina on exhale CBCT).; and if DIBH, ensure breath-hold reproducibility with visual coaching or surface tracking, and use gated imaging to verify tumor position each hold.
		High (>10 mm)	**Maximum motion management** is crucial. **DIBH** is often recommended for central tumors with large motion, as deep inspiration inflates the lungs and distances critical structures. Alternatively, use **tight gating** on the phase where the tumor is least mobile (often end-exhale). **Fiducial**	4DCT and/or breath-hold CT for planning. If using DIBH, the patient must practice breath-holds, and real-time spirometry or surface monitoring should be used; if gating, possibly combine with abdominal compression if the tumor is near the diaphragm, to reduce motion amplitude. Use real-time imaging (fluoro) during initial treatments to confirm tumor stays in gated field; use IGRT with emphasis on

			**tracking** can be considered if gating/breath-hold are infeasible, but implanting markers in central locations carries risk.	avoiding OARs: For example, verify the heart/bronchus positions relative to the target in the planned gated phase via imaging. Possibly utilize 4D-CBCT to assess motion each session.
Central	Medium (3-5 cm)	Low (<5 mm)	**Consider gated or breath-hold approach** even if motion is low, due to size and location. An *ITV approach* is possible if OAR doses meet constraints, but central medium tumors often abut structures, so reducing any margin via gating is beneficial.	4DCT ITV plus tight PTV margin if no gating (as the tumor is larger, the margin needs careful evaluation); strongly consider planning a gated or DIBH variant for comparison; and use IGRT with soft-tissue matching, considering interfraction imaging of tumor/OAR position over the course (to ensure no baseline shift).
		Moderate (5-10 mm)	**Mandatory motion management: Gating or DIBH** (preferred) to control motion. ITV-alone is not	Plan with 4DCT and gating or breath-hold, and ensure the chosen method reliably yields <5 mm residual motion; use high-quality imaging

			appropriate as it would substantially increase dose to critical central structures. Follow *guidelines (TG-76)* to use motion management >5 mm.	for setup, verifying the tumor position at the gated phase each fraction (such as using 4D CBCT or a cine verify); and if the tumor moves into mediastinum at certain phases, time the beam-off accordingly.
		High (>10 mm)	**Comprehensive approach: DIBH** is ideal (stop motion and maximize lung volume) if possible; otherwise **exhale gating** with as small a window as feasible. Multiple techniques may be combined (such as compression to steady the diaphragm plus gating). If these fail, reevaluate treatment approach (consider more fractions or even	Likely obtain both a 4DCT and an inhale breath-hold CT to decide which gives best geometry (sometimes inhale hold pulls tumor away from critical structures); use all available monitoring: For example, surface tracking for breath-hold and continuous fluoro for gatin, to ensure the tumor stays within the planned margin throughout treatment; and have a low threshold to pause treatment if breathing deviates; reproducibility is key.

			internal fixation, though experimental).	
